# Supplementary material for: Are carbon emissions trading and green financial instruments synergistic? -Comprehensive quantitative research based on content analysis
Source: PLoS One. 2024 Mar 7;19(3):e0298601. doi: 10.1371/journal.pone.0298601 (PMC10919601; doi:10.1371/journal.pone.0298601)
Supplement: S1 Data — (ZIP) [file pone.0298601.s001.zip › Data/地级市Z_F工作报告.zip!/╝¬┴╓/╝¬┴╓╩╨/└┤╫╘╣1⁄2╓┌║┼í╛┬φ┐╦╩2╛▌═°í┐_2016.docx]

各位代表：

　　现在，我代表市政府向大会报告工作，请予审议，并请政协委员提出意见。

　　一、过去五年及2015年工作回顾

　　过去五年是我市应对复杂挑战、取得重大成就的五年。五年来，我们认真贯彻省委省政府和市委的一系列重大决策部署，团结带领全市人民，坚持稳中求进工作总基调，统筹稳增长、调结构、促改革、惠民生，扎实进取，奋力拼搏，“十二五”发展任务顺利完成，经济社会发生了深刻的历史性变化。

　　过去五年是综合实力显著增强的五年。全市地区生产总值、规模工业增加值、地方级财政收入年均分别增长9.2%、5.3%和6.8%（预计数，下同）；人均生产总值达到5.7万元，高出全国平均水平7500元；实施亿元以上项目1260项，固定资产投资累计突破1万亿元，年均增长16.9%；科技经费投入是“十一五”的5.1倍，年均增长49%；战略性新兴产业规模跃上千亿级台阶，民营经济成为支撑全市经济的“半壁江山”，多业并举、多元支撑的现代产业体系架构基本形成；三次产业结构调整到9.4∶46.5∶44.1。

　　过去五年是改革开放成果丰硕的五年。扎实推进重点领域和关键环节改革，一批制约发展的深层次矛盾有效破解，经济体制改革富有成效，社会体制、民主法制改革取得重大进展，生态文明、文化体制改革同步推进。开放型经济达到新水平，长吉产业创新发展示范区强力推动，十大功能区开放载体和领先发展作用明显增强，德国大众、美国沃尔玛等一批世界500强企业入驻，累计招商引资到位资金3683亿元，实际利用外资40.9亿美元。

　　过去五年是城乡面貌变化明显的五年。累计完成城建投资1294亿元，建成5座跨江大桥、146条主次街路，城市绿化美化亮化景观特征更趋鲜明，建成区面积扩大到185平方公里，新建改建各级各类公路2827公里，山水相连、布局合理、功能完善的现代化城市空间结构形态加速形成。组织实施节能减排、低碳城市、餐厨废弃物资源化利用等一批国家试点示范，生态环境治理和保护成果显著。五县（市）发展能力持续增强，特色鲜明的县城新形象进一步展现，一批省市试点镇、中心镇发展步伐加快，新农村建设取得新成效。

　　过去五年是社会民生事业全面进步的五年。累计投入610亿元，一大批民生事项和惠民工程高质量完成；城镇、农村居民人均可支配收入分别是2010年的1.71倍、1.77倍，年均增长11.3%和12.1%。累计新增城镇就业48万人，城乡养老保险制度在全省率先实现全覆盖，成为我国北方地区第一个全城“暖房子”城市。基础教育、职业教育保持全省领先，公共卫生保障水平大幅提升，文化事业和文化产业蓬勃发展，竞技体育实现奥运金牌“零突破”。社会安全稳定局面持续巩固。

　　各位代表，刚刚过去的2015年，是“十二五”收官之年。面对经济下行压力加大、困难矛盾集中显现的复杂环境，我们按照“三个率先”发展要求，精准发力，克难攻坚，较好完成了年度160项目标任务。

　　——重点指标增长稳中有进

　　全市实现地区生产总值2473亿元，同比增长6.5%；规模工业增加值810亿元，增长6%；固定资产投资2640亿元，增长14.6%；社会消费品零售总额1311亿元，增长9.5%；全口径财政收入突破300亿元，增长9.8%；地方级财政收入132.8亿元，增长1.3%；城镇居民人均可支配收入28935元，农村居民人均可支配收入11660元，与经济增长同步。

　　——重点项目工程建设力度加大

　　针对瓶颈制约因素，集中开展征地拆迁、融资、手续办理、招商攻坚活动，丰满大坝重建、聚源聚醚多元醇等一批重点项目建设加快，5000吨粘胶长丝连续纺、4万吨乙丙橡胶、万丰工业园等项目投入生产；实施城建及交通重点工程181项，火车站西广场、吉林大街中段隧道、污水处理厂二期、全民健身中心、人民大剧院等工程建成使用，长吉南线、吉哈高速、地下综合管廊等工程进展顺利。全市建设亿元以上项目552项，竣工183项；新开工3000万元以上招商项目402项，招商引资到位资金1071亿元。

　　——重点工作推进措施有力

　　（一）突出抓经济运行，稳增长收到实效。把稳工业作为稳增长核心任务，投入1.7亿元，实施一系列定向精准措施，工业经济在严重困难情况下保持平稳运行。放大“种子基金”“助保金池”规模，加强银企保合作，为247户企业解决融资需求104亿元，帮助50户企业落实倒贷资金100亿元；强化产需衔接，化纤集团本地化采购率提高到83%，地产水泥本地化销售率达到80%；加大“上争”力度，33户企业纳入省用电奖补范围和省直购电试点；推动企业挖潜增效，重点亏损大户同比减亏2亿元，52户“双停”企业恢复生产，142项重点建成项目达产达效率提高到81%。深入推进大众创业、万众创新，新建创业孵化基地8个，新增“四上”企业300户，新生成个体工商户2.8万户，民营经济主营业务收入增长10%。提升消费对稳增长的拉动作用，雾凇冰雪节、汽车及房产博览会等消费载体功能不断放大，健康养老、文化教育、医疗保健等新兴消费蓬勃发展；“雾凇号”旅游列车开通，休闲游、度假游市场持续升温，旅游总收入增长25%；电子商务快速兴起，淘宝特色中国·吉林市馆建成使用，“一网全城”用户规模持续扩大，蛟河、桦甸进入全国首批农村电商示范县（市）行列，47个“村淘”服务站建成运营，全市网络零售交易额增长65%。

　　（二）突出抓产业升级，调结构取得新进展。着力推动传统产业向中高端延伸发展，众鑫化工12万吨生物法环氧乙烷等一批重大技改项目竣工投产，一汽大众DY整车项目成功落地，吉林铁合金、吉林炭素重组和搬迁改造扎实推进。积极培育战略性新兴产业，华微8英寸智能芯片等100个项目加快建设，培育科技“小巨人”企业144户，100种新产品实现规模化生产，战略性新兴产业产值占规模工业比重上升4.3个百分点。大力提升现代服务业水平，雾凇西路服务业集聚区投入使用，哈达湾现代服务业集聚区展现新形象；九天、吉化等重点物流企业保持良好发展态势；云计算产业中心初具规模，软件服务外包业收入增速保持在40%以上；7户企业挂牌“新三板”及区域股权交易市场，引进域外金融机构3家，村镇银行实现县域全覆盖，银行业贷款余额增长15%。统筹发展现代农业，建成1803项水利设施工程，粮食产量稳定在110亿斤阶段性水平，畜牧业、园艺特产业产值分别增长7.6%、16%；新晋市级以上龙头企业28户。推动长吉产业创新发展示范区建设，32个规划编制基本完成，192项基础设施和产业项目开工；万昌、孤店子现代农业先导区建设成效明显；中新吉林食品区食品质量安全保障体系建设深入推进，吉林大米成功出口新加坡；二台子机场改扩建项目进展顺利，山河科技通用航空轻型运动飞机组装下线；职教产业园区建设加快，全省首个B型保税物流中心建成。

　　（三）突出抓重点领域，促改革扎实推进。市场体系改革不断深化，“五证合一”企业登记制度改革全面推行，办结时限由30天缩减至3-5个工作日，营业执照颁发数量增长27.1%；放开建筑领域技术服务市场，新增中介服务机构15家。国资国企改革成效明显，理顺国有企业出资关系，国资监管能力进一步提高；50户国有企业完成主辅剥离任务，国有资本入股民营企业程度不断加深。社会领域改革稳步推进，户籍制度改革深入开展，义务教育“大学区”管理试点启动，县级公立医院实行药品零差率销售，市歌舞团上市工作进展顺利。农村综合改革实现新突破，新流转土地2.7万公顷，基本完成28个乡镇土地确权任务，发放农村土地经营权抵押贷款、收益保证贷款1.86亿元。金珠、岔路河、明城列入全省扩权试点镇。

　　（四）突出抓民生事项，惠民生成果明显。总投入130亿元的111项民生实事全部完成。城镇新增就业9万人，农村劳动力转移68万人次。城市、农村低保标准分别提高到每人每月455元和每人每年2750元。2.1万贫困人口实现脱贫。全市企业退休（退职）人员养老金实现“十一连调”，年均增幅12.6%。争取国开行98亿元融资贷款，建设棚户区房屋126.8万平方米、回迁安置1.6万户。完成“暖房子”改造178万平方米。启动环卫工人工资三年提升计划，并提供免费早餐。新增养老床位2200张。新购置公交车350台。新建农村饮水安全工程158处，解决11.6万居民饮水安全问题。完成8个游园广场绿化改造，建成10个特色花卉园。城市饮用水源工程启动，松花江流域整治行动大力实施，清收还林取得重大阶段性成果。累计投入资金3.7亿元，全面铺开农村环境“六清”整治行动，广泛开展“温暖家庭、整洁楼道、和谐社区、畅通街路”创建工作，95%的行政村和小区环境面貌发生明显改变。国家公共文化服务体系示范区获批创建。高标准承办第24届中国金鸡百花电影节，城市的知名度和美誉度大幅提升。促进义务教育优质均衡发展，9个县（市）区一次性高标准通过国家评估验收。市中心医院二期、人民医院三期等一批医疗惠民项目完工，建成标准化村卫生室418所，城乡医疗保障能力进一步增强。

　　（五）突出抓关键环节，法治政府建设迈出实质性步伐。健全重大行政决策机制，市政府决策咨询委员会组建完成。

　　建立权力清单、责任清单，行政职权削减60%，为省内保留行政职权数量最少的市（州）；市级行政审批项目减少11%，非行政许可审批事项全部取消。市公共资源交易中心在全省率先试运行，企业准入和重点项目审批“单一窗口”制度大力推行。市县两级食品药品监督管理体制改革完成。“政府法治大讲堂”常态化开展，“法律六进”等活动载体效应持续增强。深入开展“三严三实”专题教育，政府行政效能、工作作风和整体形象发生了积极变化。

　　各位代表，过去五年发展成就来之不易。经济社会取得的每一个成果，城市建设发生的每一处变化，百姓生活得到的每一点实惠，无不饱含着方方面面的关心与支持，无不凝聚着全市人民的心血与智慧。在此，我代表市政府，向辛勤劳动在各个岗位的全体市民，向所有关注、支持和参与吉林市建设发展的海内外朋友，表示衷心的感谢和崇高的敬意！

　　回顾五年的奋斗历程，我们更加深刻地认识到，推进吉林市振兴发展，必须牢固树立全市一盘棋思想，坚决在市委的领导下，在市人大、市政协的支持下，紧密团结全市上下，众志成城，克难攻坚，凝聚起谋发展、促振兴的强大合力；必须坚持一张蓝图绘到底，咬定既定目标不动摇，以功成不必在我的胸襟，一以贯之，持续发力，久久为功；必须善于抓住发展的“牛鼻子”，找准突破口、选准着力点，集中力量谋大事、干实事，用重点方面和关键环节的率先突破带动全局整体跃升；必须强化务实创新作风，以踏石留印、抓铁有痕的干事状态，以披荆斩棘、破浪前行的开拓精神，不断推进各项事业向前发展。

　　同时，我们也清醒地看到，发展中还有不少的困难和问题。一是受宏观经济形势影响，部分骨干企业大幅减产，传统产业效益下滑，全市规模工业持续低位运行，实体经济下行压力仍然很大。二是项目建设谋划储备不够，一些在建项目推进不快，具有支撑性、带动性的重大项目不多，经济增长内生动力不足。三是民生改善步伐与人民群众的发展需求不相适应，在财税收入增速下滑的背景下，公共服务保障能力不足的问题愈加突出。四是政府职能转变还不到位，有的单位和个别干部服务意识不强、效率不高、抓落实不到位以及不作为现象依然存在。对此，我们一定以高度负责的精神，认真加以解决。

　　二、今后五年重点发展任务

　　“十三五”时期是全面建成小康社会的决胜阶段。尽管外部形势错综复杂，但仍处于可以大有作为的重要战略机遇期。我们要认真学习贯彻习近平总书记系列重要讲话精神，深入落实省委“三个五”战略和“五大突破”要求，以在全省率先全面建成小康社会为目标，以新一轮振兴发展为主题，以转型升级为主线，坚持改革创新，坚持“四化”联动，全力打造旅游文化名城、新型产业基地和生态宜居城市，加快建设创新吉林、法治吉林、生态吉林、开放吉林、文明吉林和幸福吉林，加速迈向富裕和谐宜居的现代化大都市。

　　经济保持中高速增长，地区生产总值年均增长7%，全口径财政收入占GDP比重达到13%；产业迈向中高端水平，三次产业比重调整到8：42：50；城乡一体化协调发展，城镇化率达到61.7%；人居环境显著改善，生态环保指标位于全省前列；人民生活质量大幅提升，城镇、农村居民人均可支配收入年均增长10%。

　　--突出特色资源优势，全力建设旅游文化名城

　　提升“吉祥天佑、林碧水秀”的城市品质，将我市建成独具魅力的雾凇冰雪旅游基地、休闲度假旅游胜地、区域性旅游中心城市和享誉全国的特色文化城市。构建旅游新优势。突出“一江一带”旅游发展核心。依托一江秀水，着力建设“一江三湖四山”景观主轴线，规划实施松花湖5A级景区等一批重大旅游工程，构建外通内联、集群发展的核心旅游圈；依托万昌--孤店子温泉带，统筹规划布局，创新温泉+养生养老、+休闲健身、+现代农业等模式，发展特色温泉产业集群，打造服务全省、辐射东北的温泉旅游产业基地。大力发展旅游产业。打造四季皆宜旅游产品，推进雾凇冰雪节、红叶旅游节等节庆活动形成产业链；加强北大壶、松花湖等冰雪设施建设，推进“全民上冰雪”，努力把“白雪”变“白银”；建设雾凇岛旅游经济区，开发八大雾凇观赏带，确立“中国雾凇之城”世界旅游品牌地位。完善旅游公共服务体系。推进智慧旅游，实施数字景区、智慧酒店、智慧旅行社等工程，建成功能齐全、方便快捷的旅游信息服务体系；培育发展旅游商品市场和特色休闲购物街，开发出一批具有浓郁地方特色的旅游商品。到“十三五”末，旅游业总收入突破1100亿元，年均增长15%以上。彰显文化软实力。深度挖掘、传承和发展夫余文化、满族文化、船厂文化，加强文物本体保护和修缮，建设一批享誉国内外的文化遗产遗迹，打造北山、玄帝观、天主教堂、清真寺等宗教朝圣祈福文化品牌，扩大中国历史文化名城知名度和影响力。推进影视制作、演艺娱乐、出版发行等传统文化产业壮大，加快发展文化创意、动漫游戏等新兴文化产业，建设一批特色优势突出的骨干文化企业和文化产品基地，构筑形成现代文化产业集群。完善公共文化服务体系，推动文化大发展大繁荣，增强先进文化对全面建成小康社会的精神引领作用。

　　--建设"6411"产业体系，全力构筑新型产业基地

　　紧扣转型升级主线，坚持创新与继承并重，着力构建具有较强竞争力和支撑力的现代产业发展新格局。培育发展六大新兴产业。发挥资源优势，抢占制高点，大力发展以山为脊、水为脉、文为魂的旅游产业，以医药医疗、养生养老为重点的健康产业，以二台子机场复航、航空产业园建设为载体的航空产业，以电子信息制造、电子商务、云计算、软件服务外包为支撑的信息产业，以特种纤维及复合材料、新型有机化工材料、新型金属材料和新型无机非金属材料为核心的新材料产业，以交通运输设备、智能装备、节能环保装备、化工机械为代表的先进装备制造产业，努力打造辐射东北、面向全国的新兴特色产业集聚区。“十三五”期间，六大新兴产业产值年均增长10%以上。巩固壮大四大支柱产业。化工产业突出“补链”，依托原料多元化拓宽产业发展空间，完善和延伸丙烯及聚氨酯、基础化工结构升级、资源综合利用、生物质化工四大特色产业链，建成国家级新型化工产业基地。汽车产业突出“上量”，以一汽大众DY整车项目建设和一汽吉林汽车升级改造为核心，加快发展两大汽车产业基地、五个汽车零部件产业园，实现整车产能100万辆，打造全省汽车产业新核心。冶金产业突出“转型”，大力发展“围钢经济”，改造炉料、拓铁优钢、提升有色，建成东北地区重要冶金产业基地。农产品加工业突出“提质”，着力打好质量牌、安全牌，发展现代农牧业和农产品深加工全产业链，建成全国重要的绿色有机食品加工基地。提升发展现代服务业。推动生产性服务业向专业化和价值链高端延伸，大幅提升与生产制造的融合度，加快发展现代物流、金融、技术研发、会展和专业服务业；推动生活性服务业向精细、便利和高品质转变，重点发展商贸、房地产及家庭服务业，实现服务业发展新的跃升。积极发展现代农业。以建设现代农业产业、生产、经营三大体系为核心，大力发展特色效益农业，万昌、孤店子先导区建成全国现代农业示范区、中国食品质量安全国际合作试验区和国家级出口食品农产品质量安全示范区，38%的乡镇率先基本实现农业现代化。

　　--提升城市功能形象，全力打造生态宜居城市

　　突出山水特色，以旧城改造和新区建设为两翼，构筑生态良好、特色鲜明、生活舒适的品质江城。优化城市空间布局。实施“北部整合、中部优化、南部彰显”发展战略。北部，统筹规划高新北区、经开区、化工园区、金珠工业区功能布局，推进产城融合、联动发展，打造产业主导的城市副中心。中部，加大旧城改造力度，成片棚户区、危旧房、“城中村”改造基本完成；哈达湾区域完成“退二进三”，成为城市转型发展新引擎。南部，控制开发强度，提升建设品质，推动永吉口前与城区对接发展，建成生态花园都市区。完备城市综合功能。新建4座跨江大桥、7座立交桥，完成东西部快速路建设，轨道交通主城区段通车，打通断头路，畅通交通“微循环”；建设高速公路227公里，实现高速“县县通”；改建农村油（水泥）路1000公里以上。建设“一江四山”城市慢行系统，形成自行车+徒步闭环式慢行通道。提升市政公用设施建管水平，建设地下管廊140公里，实现城区水洗公厕全覆盖、生活垃圾不落地机械化收集。建设数字化城市管理平台，全面提高城市管理能力。突出生态环境治理。全面加大区域生态修复治理力度，打造百里生态长廊，建设海绵城市，持续开展清收还林，全市森林覆盖率达到57%，建成全国生态文明先行示范区，坚决留给子孙后代一个天蓝、地绿、水净的美丽家园！加大改善民生力度。打赢扶贫攻坚战，在省内率先完成8.1万人口脱贫任务，确保全面建成小康社会“一个都不能少”。以建设幸福吉林为目标，进一步完善城乡就业、社会保障、住房安居、教育医疗等公共服务体系，各项民生指标排在全省前列。

　　各位代表，“十三五”发展蓝图已经绘就，这是科学指导我市转型发展的目标引领和战略重点。我们要坚持一张蓝图绘到底，从现在开始，一年接着一年抓，一项接着一项落。为全面夯实未来发展支撑，今后五年规划实施"6411"、基础设施、城镇化、社会民生等18大工程包、2000项以上重点项目，计划完成投资超万亿元。

　　三、2016年工作安排

　　按照“十三五”发展总体部署，今年主要预期目标安排是：地区生产总值增长7%，规模工业增加值增长6%，固定资产投资增长11%，社会消费品零售总额增长9.5%，地方级财政收入增长3.5%，招商引资到位资金增长12%，城镇、农村居民人均可支配收入分别增长10%和11%，节能减排完成省下达任务。

　　（一）大力发展旅游文化产业，在旅游文化名城建设上实现新突破

　　加强景点景区建设。围绕“一江一带”建设，实施雾凇岛、红石国家森林公园、拉法山国家森林公园等重点旅游项目建设，松花湖景区达到国家5A级景区标准；高标准完成万昌--孤店子温泉带规划，统筹推进神农温泉、圣德泉、紫御温泉等8个温泉产业项目建设和运营。围绕旅游文化融合发展，完善龙潭山、帽儿山文物保护旅游示范区规划，启动朱雀阁、明清船厂遗址博物馆建设，修缮吉林文庙，完成佛教文化园、玄帝观、吉林水师营博物馆建设项目，着力建成一批精品旅游文化景观。

　　打造特色旅游文化品牌。组织好中国首届道教文化艺术节、非物质文化遗产展示、端午节龙舟赛等重大节庆赛事活动，积极发展节庆赛事特色品牌。抓住北京承办2022年冬奥会契机，启动“全民上冰雪”计划，规划建设雾凇室内体验馆，争创国家级冰雪训练比赛基地，做大以赏凇、滑雪、浴温泉为主题的冰雪旅游品牌。培育国家级休闲农业与乡村旅游示范县2个、节点镇7个、示范村10个、星级示范企业10户，打造具有乡土风情和地域特色的乡村旅游品牌。整合旅游资源，精心策划覆盖全域、全时节的特色旅游线路，创建“国家全域旅游示范区”品牌。

　　提升综合服务水平。以改善吃、住、行、游、购、娱条件为重点，加快佰金翰、维也纳等一批四星级以上酒店建设进程，完成青山连接线、松滨东路改造等景区道路工程。启动“互联网+旅游”平台建设，发展网上查询、预订、支付、投诉等服务，建设游客服务中心。完善旅游商品体系，加强研发设计、生产加工和销售网络建设，统一开发运作东北三宝、浪木根雕、乌拉贡品等特色旅游商品。充分发挥人民大剧院、全民健身中心等演艺载体功能，策划开展大型商业演出，推出一批精品驻场演艺节目。加强在国内主流媒体的宣传推介，开展影视拍摄、文学创作、艺术写生等主题活动，不断扩大我市对外影响力。

　　（二）着力构建现代产业格局，在新型产业基地建设上实现新突破

　　抓好新兴产业培育。健康产业：发展健康医药产业，促进海王药业等3户医药企业、长白山蒺藜脑通胶囊等6个重点医药项目达产达效，快速形成医药保健产业集群；发展健康“养老”“养小”产业，着力建设长吉养老社区、中西医结合医院养老康复中心等一批医养结合型专业机构，扶持社会资本建设提升一批月子中心、母婴会所和早教机构，形成专业化、一体化健康服务体系；发展健康疗养产业，依托温泉资源，建设四季祥康温泉社区、福郡明珠小镇等一批康体疗养项目，打造“温泉+健康”疗养保健新模式。航空产业：启动二台子机场改扩建工程，推进飞行区、航站区等基础设施建设，抓好吉航民机维修、福航航空学院等重点项目。信息产业：加快通信基础设施建设，4G基站比例提升到40%以上；推进省、市联通数据中心项目，拓展江南数据中心商业化运营领域，大仝软件外包基地建成使用，国家“两化”融合试点企业发展到10户。新材料产业：集中开发高性能聚乳酸纳米复合材料等新产品，加快生物基降解复合材料、车用耐高低温塑料光纤等科技成果产业化步伐，丁苯橡胶、乙丙橡胶、1.3万吨生物长丝等一批项目年内形成有效产能。先进装备制造产业：抓好华微新型电力电子器件基地、森远筑路机械制造基地建设，鼓励永大、龙鼎等企业开发高端产品，支持昊宇石化、明阳风电等企业扩大产能，推进旭峰科技3D打印、赛金得冲压焊接线、吉煤煤机精密制造等一批重点项目成规模、增效益。

　　抓好支柱产业升级。围绕“补链”，构建丙烯及聚氨酯产业链，启动康乃尔60万吨甲醇制烯烃项目，弥补乙烯、丙烯等基础化工原料缺口，带动下游吉神30万吨环氧丙烷、聚源40万吨聚醚、众鑫化工和博海生化18万吨环氧乙烷项目产能释放；完善基础化工产业链，开展中油吉化汽油国V升级改造，实施乙二醇改产环氧乙烷项目，推进原料药、阿巴卡韦中间体等产品提能扩产；拓展资源综合利用产业链，启动大地化工硫磷钛循环利用项目，推进瀚禹化工二萘酚等综合利用项目，促进资源可循环、再利用；发展生物质化工产业链，深化与中粮、绿能等公司合作，加快建设生物质颗粒燃料、秸杆制沼气等生态环保示范项目，下力气在秸杆转化上取得实质性突破。围绕“上量”，提高整车产能，全力推进一汽大众DY整车项目上半年开工，一汽吉林汽车微卡合作项目达到2万辆生产能力；带动汽车配套产业发展，着力引进大众系列核心配套企业，加快万丰奥威50万套铝镁合金汽车结构件、奥贝球铁二期等项目建设，吉星1500万条半钢子午线轮胎、龙山车桥主减速器等产品实现批量生产。围绕“转型”，启动吉林铁合金、吉林炭素异地搬迁，推进80万吨铁合金冶炼、12万吨碳素及制品等技改升级项目合作；巩固建龙300万吨钢铁生产能力，支持钰玲珑等管件企业、北工等钢构企业扩大产能，加强与杭箫钢构等国内知名企业合作，提高钢材就地转化率。围绕“提质”，推动新加坡狮诚婴幼奶粉生产、德生牧业肉鸡屠宰加工等重点项目尽快落地，扶持东福、宇丰等龙头企业做大做强，进一步提高农产品精深加工能力。

　　抓好服务业跃升。促进服务业扩大规模、提档升级，服务业增加值占GDP比重达到45.4%。发展现代物流业。加快增益吉林内陆港、煤炭精洗及物流园等建设进度，完善保税物流中心功能，推进博宇医药物流园区运营达效，危化品运输服务中心启动运行。发展现代金融业。以建设省级金融改革创新试验区为引领，新引进域外金融机构2家、设立村镇银行2家、新增上市和挂牌企业10户；支持银行业扩大信贷投放规模，力争实现重点项目贷款700亿元、中小微企业贷款410亿元。发展商贸流通业。推进东北亚果蔬批发、中凯家居建材港等交易市场建设，促进亚琦国际物流商贸城、百业国际五金汽配城形成集聚效应，加快实施万达城市广场、河南街商业中心等区域商贸项目，支持福源馆、新兴园等老字号企业向连锁化、集团化方向迈进。发展电子商务。深化与阿里巴巴、京东等知名电商的战略合作，抓好跨境电子商务园区、淘宝特色中国·吉林市馆扩建等项目，扩大“一网全城”等本地电商的区域影响力，推动电子商务进农村、进企业、进社区，全市网络零售交易额增长30%以上。发展房地产业。优化开发结构，加快房屋产权办理，打通回迁安置与商品房市场通道，发展房屋租赁市场，进一步激活二手房市场，促进房地产业平稳健康发展。

　　抓好现代农业发展。打造现代农业样板。加快万昌、孤店子两个现代农业先导区建设，实施好一批农田灌区基础设施和农业产业化项目，落实好29项现代农业指标体系；促进中新吉林食品区发展提速，新建续建生猪产业化等10个产业化项目，国家区域食品和农产品检验检测中心投入使用，争取更多产品进入以新加坡为主的国际市场。提升农业综合生产能力。推广17项粮食增产技术，建设高标准基本农田6987公顷，农业综合机械化率提高到74.5%；壮大芦笋、食用菌等10大特色产业，抓好50个特色标准园区建设；完成无疫区建设任务，新改扩建规模化、标准化养殖场210个；整合松花湖渔业资源，全力塑造吉林渔业发展新形象。加强农业品牌建设。打响“中国粳稻贡米之乡”品牌，扩大长白山黑牛、吉林花猪、黄松甸木耳、长白山林蛙等品牌知名度和市场占有率，新增“三品一标”认证农产品22个。

　　（三）着力完备综合服务功能，在生态宜居城市建设上实现新突破

　　加强城市建设管理。提升城市承载能力。建设城建重点工程147项，完成投资138.6亿元。实施九江大路西段、经四路等19项功能区基础设施工程，开工哈达湾南北干线等17条地下综合管廊，启动城市轨道交通融冰除雪实验项目，新建续建长吉南线、九站高速出口改造、白山大桥、松江南路、深圳东路等路桥工程，完成26项路桥维修改造任务；高水准美化绿化亮化沿江两岸等区域，完善提升人民广场、世纪广场等配套设施。提高城市管理精细化、网格化、常态化水平。理顺城市管理体制，全面实施综合执法，构建“大部门制”城管执法格局；启动智慧城市建设，总结推广丰满区网格化城管试点经验，搭建数字化城管平台；深入开展“温暖家庭、整洁楼道、和谐社区、畅通街路”创建活动，继续争创全国文明城市。梯次推进城镇化建设。以国家新型城镇化试点建设为统领，拓展五县（市）发展空间，吸纳农村转移人口，打造次级中心城市发展极；充分发挥金珠、北大湖等示范城镇带动作用，完善城镇规划设计，实施“三区三改”，加快建设一批各具特色的小城镇。

　　突出生态环境建设。让“水更清”，加大松花江流域水污染防治力度，开工龙潭川、北大沟、东大沟综合整治工程，改造治理远大河、丰满沟水体，实施市污水处理厂一期提标、二次供水改造等水污染防治项目，加快松花江生态长廊、温德河湿地公园建设。让“天更蓝”，建立重污染天气应急响应机制，实施9户重点工业企业烟气脱硫工程改造，工业企业烟粉尘排放达标率达到95%以上；完成城区禁燃区90%燃煤小锅炉整治任务，推动大中型餐饮企业油烟治理达标，淘汰黄标车1000辆，实现秸秆综合利用20万吨以上。让“地更绿”，做好晨鸣纸业搬迁后污染场地修复、松花湖近湖区生态修复工作，开展好污染土壤修复与综合治理试点示范；启动农药化肥零增长行动；实施矿山整治工作；巩固清收还林成果，再还林50万亩以上。让“农村更洁净”，提升“六清”标准，深入开展农村人居环境整治行动，集中创建10个省级美丽乡村、62个样板村。

　　保障和改善民生。坚持守住底线、突出重点、尽力而为、量力而行，最大程度地让人民群众共享改革发展成果。完善社会保障体系。开展精准扶贫攻坚，实现贫困村和贫困人口脱贫任务“双过半”。落实低保“补差式”救助，城乡低保标准分别达到上年度城镇、农村居民人均可支配收入的20%和30%。深化医保支付制度改革，城镇职工和居民门诊慢性病、重大疾病报销病种扩大到26种。稳妥推进机关事业单位养老保险制度改革，适度提高退休人员基本养老金和城乡居民基础养老金标准。改善居民生活条件。改造城市棚户区和危旧房7300户、50万平方米，分配公共租赁住房2800套、发放租赁补贴7500户。启动松江路、滨江路25公里城市慢行系统；新购公交车150台，新开、调整公交线路20条；维修改造89条小街小巷和43个小区配套设施；新建改建农村公路100公里。铺设燃气配套管网15公里，更新供热管网110公里，改造9条雨污排水管线，新建改造水洗公厕69座，启动建设餐厨废弃物处理厂。抓好“菜蓝子”工程，繁荣稳定市场供应。发展社会事业。努力改善办学条件，新建改建公办幼儿园45所，改造义务教育薄弱校38所，实施“宽带网络校校通”建设。加大医疗惠民力度，免费为1万人提供心脑血管疾病高危筛查，实施市第六医院和市结防所等改建项目，吉林卫校异地新建工程年内竣工。积极开展国家公共文化服务体系示范区创建工作，启动市广电传媒中心、美术馆建设，组织开展广场文化活动周、公益电影放映等活动，进一步活跃城乡居民文化生活。实施冰雪奥运冠军培育计划，开展系列体育健身活动，承办好CBA、全国高山滑雪赛等大型体育赛事，打造东山体育运动核心圈。坚决维护社会安全稳定。完善安全生产长效机制，严格落实安全生产目标责任，强化重点领域专项整治，坚决杜绝重特大安全事故发生。防范和打击非法集资，确保不发生区域性金融风险。加强依法治访，全面落实事涉单位主体责任和属地稳控责任，强化“五位一体”包保措施，加大“事要解决”力度，妥善处理好人民群众的合理诉求。加快智能交通和视频监控系统建设，健全立体化社会治安防控体系，进一步提升社会治理科学化水平。加强国防动员和人民武装工作，促进军地融合协调发展。

　　（四）积极推进改革创新，为城市转型发展提供强大动力支撑

　　深化重点领域改革。以供给侧结构性改革为牵引，落实一批重要改革事项。推进国资国企改革。全面完成市属经营性国有资产集中统一监管；分类做好国企改革工作，调整优化国有资本布局结构，打造一批具有较强竞争力的企业集团；推动金控集团、水务集团、歌舞团等企业改制上市，加快国有资本证券化进程；规范董事会建设，推进外派监事会设立，强化国有企业监督。推进市场体系改革。实行统一的市场准入制度，编制完成负面清单，实现企业注册全程电子化；进一步放开建设领域技术服务市场；科学调整居民污水处理费，推进民用天然气阶梯价格改革，深化供热及交通、旅游相关价格改革。推进投融资改革。整合国有资源，按照成熟一支设立一支的原则，加快产业发展基金组建；扩大PPP合作成果，力争更多项目进入政府和社会资本合作范畴。推进社会领域改革。继续实施义务教育“阳光入学”，调整优化学校布局，促进教育优质均衡发展；扎实做好公立医院改革和基层医疗卫生机构综合改革，鼓励社会资本采取股份制合作方式参与公立医院发展建设，推行分级诊疗就医模式。推进农村综合改革。启动国有林场、供销社改革，继续开展土地收益保证贷款和土地经营权抵押贷款工作，土地流转面积达到12万公顷，完成33个乡镇土地确权任务。

　　深入实施创新驱动。强化科技创新。实施100项科技攻关及产业化项目，在新材料、通用航空、信息技术等重点领域实现新突破；深化产学研协同创新，与中科院、吉林大学等域外高校院所合作筹建产业技术研究院，推动驻吉高校与本地市场深度融合，培育科技“小巨人”企业200户；高起点规划科技创新城，启动高新北区核心区一期项目建设。推进大众创业、万众创新。依托巨邦软件园、电商产业园、科技大市场等载体，大力发展市场化、专业化、集成化、网络化的“众创空间”，建设10个创新创业示范基地，带动城镇新增就业9万人。深入实施“万名创业者、万名小老板”工程，全面开展“互联网+小微企业”行动，民营经济主营业务收入增长8%。实施江城英才创新创业扶持计划，培育、引进一批高层次创新创业人才和“双创”团队；完成职教产业园区主体工程建设，开展现代学徒制试点，积极探索职业教育与产业融合发展模式，着力培养一大批适应产业发展需要的技术技能型人才。

　　（五）加快建设法治政府，为城市转型发展营造高效政务环境

　　坚持科学理政，推进政府职能转变。施行市政府重大行政决策程序暂行规定和咨询工作规则，突出发挥好市决咨委智囊作用，进一步提高政府依法决策、科学决策、民主决策水平。以减程序、减费用、减时限为重点，全面清理行政审批、公共服务和中介服务，完成网上政务大厅建设，启动重点投资项目在线审批“单一窗口”试点。开通城市门户网站，搭建政务云平台。推进公共资源交易管理体制改革，建立与县（市）互联互通的全过程电子化交易平台。实施政府重点工作目标网格化推进和扁平化管理，确保政府各项工作落到实处、取得实效。

　　坚持依法行政，切实维护法律权威。严格按照权力清单、责任清单履行职权，做到“法无授权不可为、法定职责必须为”，坚决纠正不作为、乱作为。严格规范公正文明执法，全面推行行政执法责任制，落实重大行政处罚备案制度，切实提高行政执法质量和水平。完善国家工作人员学法用法制度，办好“政府法治大讲堂”，落实“谁执法谁普法”责任制，把依法行政落实到政府工作全过程。

　　坚持从严治政，持续推进作风建设。自觉接受人大及其常委会的法律监督、工作监督和政协的民主监督，主动接受司法、媒体和公众监督。全面实施财政预决算、“三公”经费、征地拆迁补偿等重点领域的信息公开，深化公共企事业单位办事公开，推进公共资金、国有资产、国有资源和领导干部履行经济责任审计全覆盖。巩固群众路线教育实践活动成果，自觉践行“三严三实”，持续推进政府系统党风廉政建设，努力建设一支勇于作为、敢于担当、勤政为民、清正廉洁的人民公仆队伍。

　　为有效落实全年任务，按照“十三五”18大工程包安排，今年计划实施5000万元以上项目500项，其中，"6411"产业项目工程包310项，基础设施项目工程包100项，社会民生项目工程包45项，城镇化项目工程包10项，生态环保项目工程包10项，其它25项。发挥招商引资对项目建设的支撑作用，围绕现代产业体系建设，瞄准国内外知名企业和战略投资者，积极“走出去”“请进来”，开展精准务实招商，推进招商引资不断取得实效。年内谋划包装5000万元以上招商项目500项，新开工重点招商项目300项，招商引资到位资金1200亿元。

　　各位代表，新一轮振兴发展的征程已经开启。让我们在省委省政府和市委的正确领导下，紧紧团结和依靠全市人民，以更高的标准、更实的作风、更大的担当，埋头苦干，奋力作为，加快建设“六个吉林”，向着全面建成小康社会的宏伟目标昂首迈进！
